# Nitrogen-doped graphene films from chemical vapor deposition of pyridine: influence of process parameters on the electrical and optical properties

**DOI:** 10.3762/bjnano.6.206

**Published:** 2015-10-14

**Authors:** Andrea Capasso, Theodoros Dikonimos, Francesca Sarto, Alessio Tamburrano, Giovanni De Bellis, Maria Sabrina Sarto, Giuliana Faggio, Angela Malara, Giacomo Messina, Nicola Lisi

**Affiliations:** 1 ENEA, Materials Technology Unit, Surface Technology Laboratory, Casaccia Research Centre,Via Anguillarese 301, 00123 Rome, Italy; 2 Istituto Italiano di Tecnologia, Graphene Labs, I-16163 Genova, Italy; 3 ENEA, Fusion Technical Unit, Lab. of Nuclear Technologies, Via Enrico Fermi 45, 00044 Frascati (Rome), Italy; 4 Research Center on Nanotechnology Applied to Engineering of Sapienza (CNIS), SSNLab, Sapienza, University of Rome, P.le Aldo Moro 5, 00185 Rome, Italy; 5 Dipartimento di Ingegneria dell'Informazione, delle Infrastrutture e dell'Energia Sostenibile (DIIES), Università “Mediterranea” di Reggio Calabria, 89122 Reggio Calabria, Italy

**Keywords:** carbon, electrical conductivity, nitrogen doping, optical conductivity, transparent conductor

## Abstract

Graphene films were produced by chemical vapor deposition (CVD) of pyridine on copper substrates. Pyridine-CVD is expected to lead to doped graphene by the insertion of nitrogen atoms in the growing sp^2^ carbon lattice, possibly improving the properties of graphene as a transparent conductive film. We here report on the influence that the CVD parameters (i.e., temperature and gas flow) have on the morphology, transmittance, and electrical conductivity of the graphene films grown with pyridine. A temperature range between 930 and 1070 °C was explored and the results were compared to those of pristine graphene grown by ethanol-CVD under the same process conditions. The films were characterized by atomic force microscopy, Raman and X-ray photoemission spectroscopy. The optical transmittance and electrical conductivity of the films were measured to evaluate their performance as transparent conductive electrodes. Graphene films grown by pyridine reached an electrical conductivity of 14.3 × 10^5^ S/m. Such a high conductivity seems to be associated with the electronic doping induced by substitutional nitrogen atoms. In particular, at 930 °C the nitrogen/carbon ratio of pyridine-grown graphene reaches 3%, and its electrical conductivity is 40% higher than that of pristine graphene grown from ethanol-CVD.

## Introduction

Transparent conductive electrodes (TCEs) are an indispensable component of many kinds of electronic devices, such as displays, touch-screens, light emitting diodes, and solar cells [1–8]. Since its discovery, graphene was proposed as an ideal material for TCEs thanks to its transparency and superior electrical conductivity [9–10]. To date, graphene films have been produced through a multitude of different techniques and used to fabricate devices. However, the achieved electronic characteristics still need further improvement for a fruitful application in consumer electronics [11–12]. Although the highest electron mobility is usually reached in mechanically exfoliated graphene crystals [13], the most suitable route for the production of high-quality graphene for electronics is probably chemical vapor deposition (CVD). By this technique it is possible to produce graphene with large grain sizes and high crystalline quality over large areas [14]. Nonetheless, the sheet resistance of most CVD-graphene films (even in single-crystal form) still falls short of the requirements for TCEs [14–16].

The impact that graphene will have on many fields of electronics ultimately depends on the real properties it will be able to provide. Thus, doping of graphene is currently considered a promising way of enhancing its carrier density and improving the electrical conductivity to satisfy the requirements of various electronic applications [16]. Nevertheless, the product of electron mobility and dopant concentration generally remains constant, and thus there is a limit to the achievable improvement in electrical conductivity by this approach [17]. Besides, the electron mobility itself can be greatly affected by the presence of substitutional atoms (which are a kind of lattice defects, such as vacancies, and grain boundaries) [18].

Graphene can be doped through surface proximity by layering it with other materials (such as metals [19], polymers [20], atoms [21] and molecular functional groups [22]) that alter its electronic properties and can also, in principle, open a bandgap, giving graphene semiconductor properties. In an alternative way, graphene can be doped by substitution, i.e., by inserting heterogeneous atoms into the lattice. When using CVD, the choice of a carbon-based precursor containing specific atoms or groups can give rise to direct, single-step growth of doped graphene. In contrast to proximity doping, substitutional doping modifies the crystal lattice of graphene but generally preserves the chemical inertness of the material.

Due to its size, nitrogen is one of the few atoms (along with boron) that can fit within the graphene lattice [23]. When bound to carbon atoms sharing four valence electrons, nitrogen should ideally confer n-type doping to graphene due to the availability of an extra electron. Doping with nitrogen might also confer useful chemical properties to graphene, e.g., rendering it catalytic to oxygen reduction reactions [24] or enhancing its lithium intercalation properties for battery applications [25]. Nitrogen doping was originally achieved ex situ by the post-growth treatment of pristine CVD graphene in ammonia gas [26]. However, few attempts have been made of directly growing nitrogen-doped graphene onto metal foils by CVD using N-containing precursors. On platinum surfaces, nitrogen-doped carbon films were grown below 500 °C with acetonitrile and below 700 °C with pyridine [27–28]. On copper foils, nitrogen-doped graphene was grown with dimethylformamide vapor at 950 °C [29].

In this paper, we grew graphene films by CVD of pyridine on copper surfaces, evaluating the occurrence of doping by substitutional nitrogen atoms in the films. Several groups recently started to produce graphene using liquid carbon precursors such as ethanol (instead of hydrocarbon gases, such as methane) because of their effectiveness, safety and low-cost. Pyridine (C_5_H_5_N) is a liquid precursor akin to ethanol (C_2_H_5_OH). The main difference between the two is that ethanol is generally used to make pristine graphene while pyridine can be used to form nitrogen-doped graphene. Our group recently showed that highly-crystalline, pristine graphene can be produced by ethanol-CVD on copper above 1000 °C [30–31]. However, it has been reported that below 900 °C the graphene films grown by ethanol-CVD showed some evidence of oxidation [32]. Ethanol is known to decompose during CVD into oxygen-carrying and hydrocarbon molecules or radicals: Below a certain temperature, some of the oxygen atoms or groups might bind to defects, grains or edges [33]. Likewise, in the case of pyridine-CVD, the temperature can have a profound effect on the insertion of nitrogen atoms into the graphene lattice (and hence on the doping level). Pyridine decomposes mainly into hydrogen, acetylene (C_2_H_2_) and hydrogen cyanide (HCN) [34], which is the compound expected to be at the basis of the heterogeneous doping of graphene. The dissociation of HCN and the interaction of the dissociated species with the copper surface (and with the forming graphene clusters) can be strongly influenced by the CVD parameters (i.e., temperature and vapor composition). The growth of nitrogen-doped graphene by CVD of pyridine was recently demonstrated at 1000 °C [35]. To date however, there are no systematical studies on the effect of temperature and hydrogen flow in pyridine-CVD. Therefore, we here explored a wide range of CVD process parameters (930–1070 °C for temperatures, 0–100 sccm for H_2_ flow in the vapor mixture) to assess their effect on the graphene properties. In order to better evaluate such effect and assess the occurrence of nitrogen doping, we further run a systematic comparison with the graphene films made by ethanol-CVD under analogous conditions [30].

## Results and Discussion

### Spectroscopic analyses of the graphene samples

The graphene films grown by pyridine for 10 min at different temperatures (930 °C, 1000 °C and 1070 °C) with two different hydrogen flows (1 and 100 sccm) were transferred on Si/SiO_2_ (300 nm) and analyzed by micro-Raman spectroscopy. At the optical microscope, the graphene films showed the typical features of those grown by other carbon precursors (such as methane and ethanol) on commercial copper foils. They are overall uniform and have some darker areas running along parallel stripes, which are graphene grains developed at sites of secondary nucleation induced by the lamination process undergone by the copper foil [36–37].

**Figure 1:**
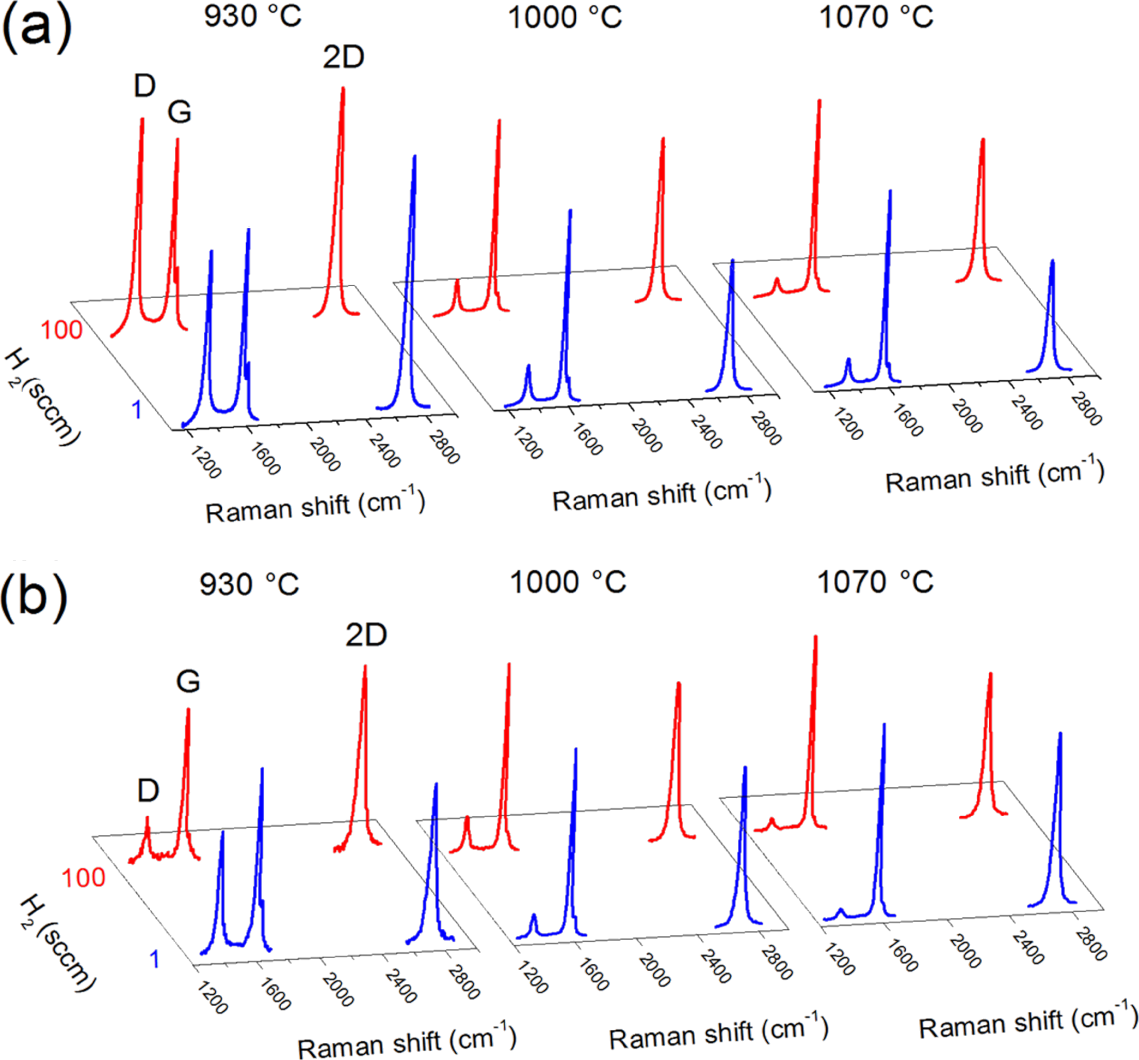
(a) Raman spectra of graphene grown by pyridine, at different temperatures and hydrogen flows. (b) Raman spectra of graphene grown by ethanol.

Figure 1 shows the spectra of graphene films grown by CVD of pyridine at different temperatures and with two hydrogen flows. The spectra of graphene films grown by CVD of ethanol (10 min, with 1 and 100 sccm of hydrogen) are also reported for comparison. The results of the peak fitting (peak features and intensity ratios *I*_D/G_, *I*_2D/G_, *I*_D/D’_) are listed in Supporting Information File 1 (Table S1 and Table S2).

In all the Raman spectra, the typical G and 2D peaks of graphene (centered at ≈1585 cm^−1^ and ≈2700 cm^−1^, respectively) are present. The D and D’ peaks (≈1350 cm^−1^ and ≈1620 cm^−1^, respectively) are also visible and usually indicate the presence of defects in the graphitic lattice such as disordered carbons, edges and wrinkles [38–39]. Different atoms inserted in the graphitic lattice, such as substitutional nitrogen, can as well act as defects, in principle contributing to the D and D’ signal.

In Figure 1 it appears that above 1000 °C the films have overall analogous Raman features. At 930 °C instead, the D and D’ peaks become particularly intense, especially in the case of pyridine graphene.

The positions of the peaks and their intensity ratios (*I*_D/G_, *I*_2D/G_, *I*_D/D’_) are reported in Table S1 (Supporting Information File 1). The *I*_2D_/*I*_G_ intensity ratios can be used to estimate the film thickness: single-layer and bi-layer graphene are characterized by *I*_2D_/*I*_G_ > 1, whereas multilayer graphene typically has *I*_2D_/*I*_G_ < 1 [40].

For pyridine-CVD, temperatures above 1000 °C seem to favor the growth of multilayer graphene (*I*_2D_/*I*_G_ ranging from 0.6 to 0.86), whereas at 930 °C the films seem to be composed of 1 to 3 layers (*I*_2D_/*I*_G_ between 1.29 and 1.55). This situation is in line with graphene grown through ethanol-CVD (see Table S2, Supporting Information File 1). As for the effect of hydrogen flow, at all the three temperatures hydrogen appears to have a weak influence on the peak positions and on the *I*_D_/*I*_G_ ratio. This is in contrast with what usually reported for ethanol-CVD, where the role of hydrogen was found to be crucial in determining the crystalline quality and defect density of the graphene films [30–31].

Concerning the D and D’ peaks, the highest intensities are observed at the temperature of 930 °C (*I*_D_/*I*_G_ intensity of 0.92–1.16, see Table S1, Supporting Information File 1), possibly indicating the occurrence of nitrogen doping. Table S1 also reports the values of the graphene domain size *L*_a_ evaluated according to the relation *L*_a_ = (2.4 × 10^−10^) λ^4^(*I*_D_/*I*_G_)^−1^ (with λ being the Raman exicitation wavelength and *I*_D_/*I*_G_ integrated intensity ratio) [41]. The low *L*_a_ values obtained at 930 °C indicate a low average inter-defect distance; however the narrow full width half maximum (FWHM) of ≈28 cm^−1^ seems to point out that these highly dense defects should belong to a single type [39]. Comparing the 930 °C values reported in Table S1 (Supporting Information File 1) for pyridine-CVD to those in Table S2 (Supporting Information File 1) for ethanol-CVD, the *I*_D/G_ ratios of the samples grown by pyridine (0.92 at 1 sccm H_2_ and 1.16 at 100 sccm H_2_) are consistently higher than those of the ethanol samples (0.68 at 1 sccm H_2_ and 0.25 at 100 sccm H_2_). This *I*_D_/*I*_G_ increment for pyridine-CVD might be thus ascribed to the insertion of nitrogen in the graphene film.

It was shown that the *I*_D_/*I*_D’_ intensity ratio can be used experimentally to get information about the nature of defects in graphene [42]. The *I*_D_/*I*_D’_ ratio is found to be maximal (about 13) for defects associated with sp^3^ hybridization. It decreases for vacancy-like defects (about 7), and reaches a minimum for boundary-like defects (about 3.5). In the spectra of the pyridine-CVD films grown at 930 °C, the values of *I*_D_/*I*_D’_ (Table S1, Supporting Information File 1) are always smaller than 4.5. This seems to suggest the occurrence of boundary-like defects, which would be linked to the presence of nitrogen atoms, coordinated either in the lattice or along grain boundaries.

**Figure 2:**
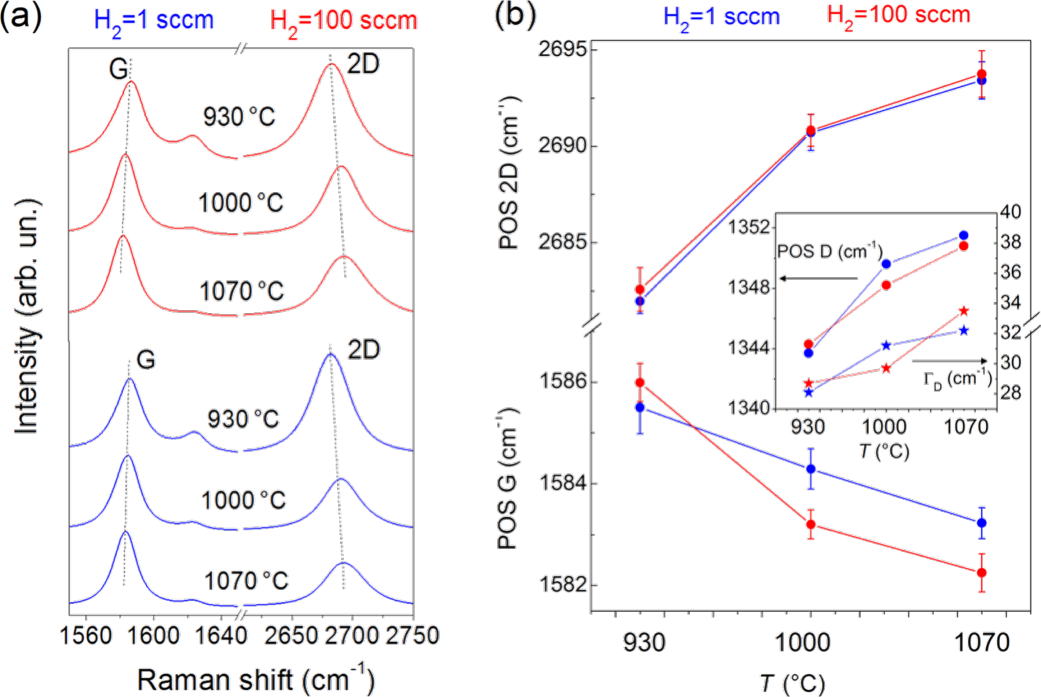
(a) Raman spectra of graphene samples grown with 1 and 100 sccm of hydrogen flow,(b) position of the G and 2D peaks vs temperature (inset shows the shift of the D position and its corresponding Γ variation).

In Figure 2a, the influence of temperature and hydrogen flow on the G and 2D Raman bands is further investigated. When lowering the CVD temperature, the position of the G peak is observed to slightly upshift, while the 2D peak position downshifts. Raman spectroscopy can monitor doping in graphene [43]. The G and 2D bands of graphene respond to doping and in particular, an upshift of the G band usually demonstrates the occurrence of doping (holes or electrons) in graphene films, while the shift of the 2D band indicates the type of doping (n-type for a downshift, p-type for an upshift) [44]. The D band was also reported to vary with doping [45]. The authors reported that its frequency increases in the case of hole doping and decreases for the electron one, whereas, its FWHM increases at low carrier density doping (by both electrons and holes), but decreases for heavy electron doping [45]. In our samples, upon lowering the temperature, the D peak narrows and its frequency position decreases by about 7 cm^−1^ (inset in Figure 2b). All these results combined strongly suggest the occurrence of n-type doping, likely due to the introduction of nitrogen atoms into the carbon lattice.

**Figure 3:**
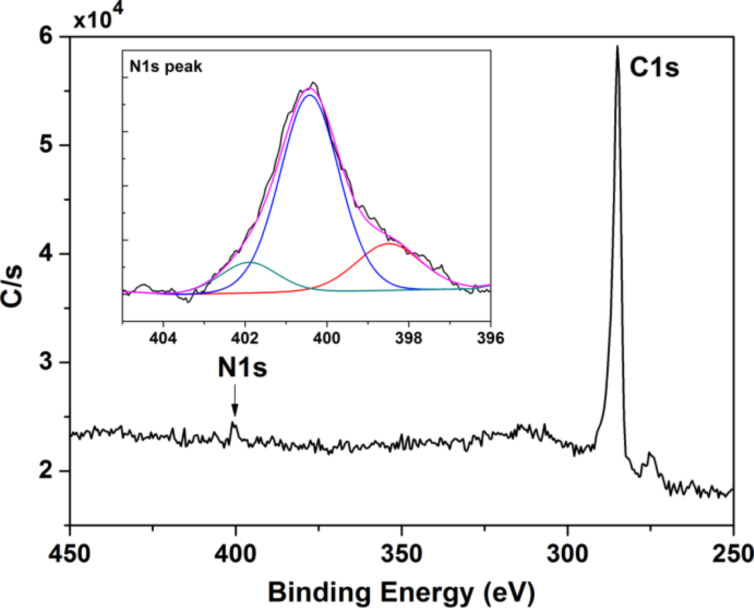
XPS spectrum of graphene grown by pyridine at *T* = 930 °C, 1 sccm H_2_, 10 min. The N1s peak is shown in the inset with a N/C ratio of about 2.9%.

Samples grown at 930 °C were further analyzed in detail by XPS (Figure 3). The wide spectrum in Figure 3 is typical of a pristine graphene transferred on Si/SiO_2_, with the exception of an additional nitrogen component. The N1s peak, laying between 397–403 eV, was closely investigated. The peak is asymmetric and can be fitted with three components centered at 398.5, 400.4, 401.9 eV, which can be ascribed to pyridinic, quaternary (graphitic) [46] and oxidized N groups, respectively [47–48]. Pyridinic and quaternary are two of the three most common bonding configuration (both sp^2^ hybridized) for nitrogen within a carbon lattice (the third being pyrrolic, the only one that is sp^3^ hybridized) [28]. Specifically, N is in pyridinic configuration when it bonds with two C atoms sitting at an edge or a defect of the lattice (the N here contributes one electron to the π system); in the quaternary configuration, instead, the N atom is inserted in the hexagonal ring substituting a C atom (and thus contributing two electrons to the π system). The component assigned to oxidized N groups has been already observed in nitrogen-doped graphene and carbon nanotubes [49–51]: in this case, the N atom usually bonds with one O and two C atoms.

It should be noted that the curve deconvolution of the N1s spectra does not seem to show trace of pyrrolic N, i.e., N atoms contributing two p electrons to the π system (e.g., the kind of coordination an N atom has in the five-membered C ring of the pyrrole molecule). When present, the peak from pyrrolic N is expected to be located in between the pyridinic and quaternary peaks, at a distance of about 1.1–1.2 eV in binding energy from each of them [52]. In our XPS analysis, the quaternary peak is shifted by 1.9 eV form the pyridinic peak (at 398.5 eV), in perfect agreement with similar experiments [53], and no further peak is revealed in between them. As for the oxidized N peak, this is shifted by 1.5 eV from the quaternary peak, as already reported [51]. The absence of the pyrrolic sp^3^-hybridized component in XPS is found to be in agreement with the measured Raman *I*_D_/*I*_D’_ ratio, which points only to sp^2^, boundary- or point-like defects.

The samples grown at 1000 and 1070 °C were also investigated by XPS. The 1000 °C sample showed evidence of nitrogen, but the signal-to-noise was not high enough to reproducibly assess the N/C content and/or ascertain the binding components. In the 1070 °C samples it was not possible to univocally determine the presence of nitrogen. Overall, the XPS spectra at each temperature were observed to be not affected by the hydrogen flow, as the Raman analysis.

### Characteristics of the films as transparent conductive electrodes

#### Sheet resistance and optical transmittance

**Figure 4:**
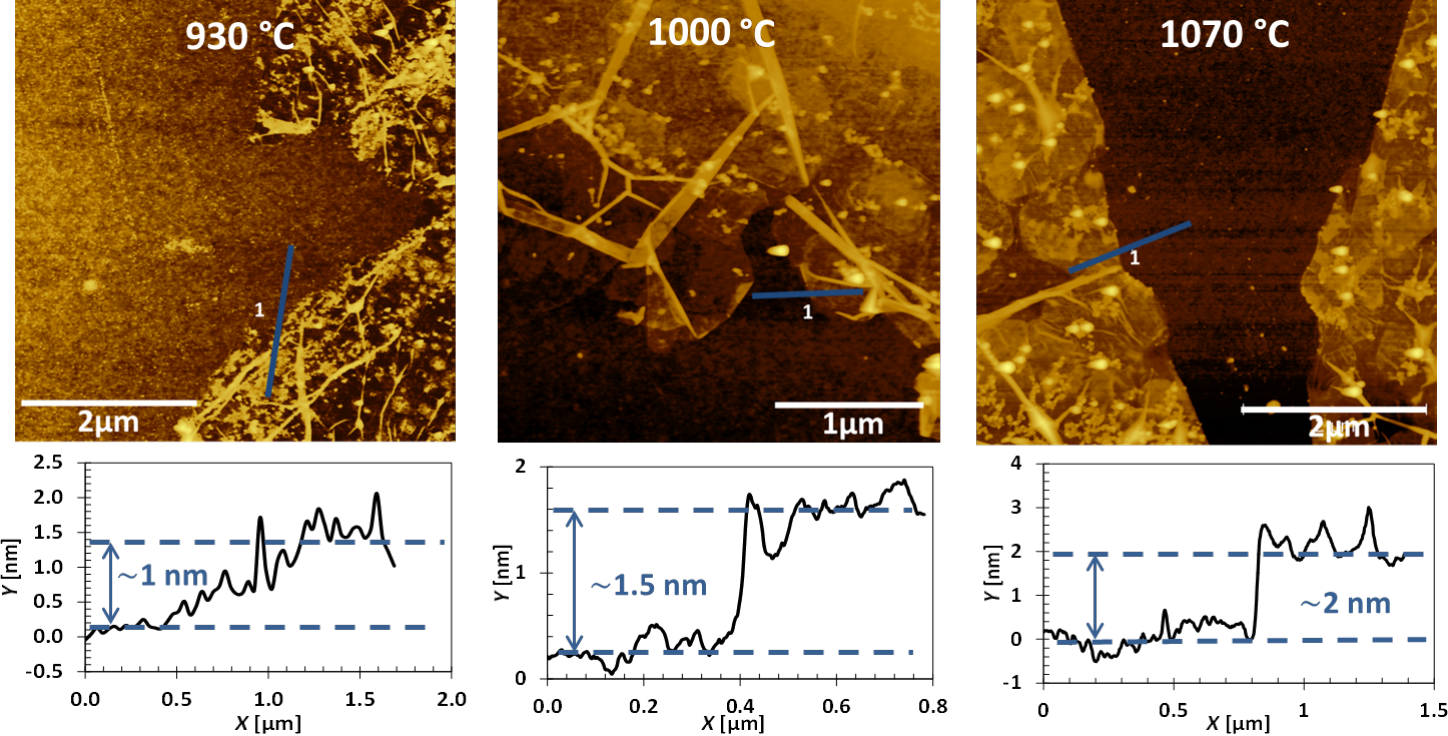
AFM micrographs of graphene films grown by pyridine-CVD for 10 min at 930, 1000 and 1070 °C. Line profiles, providing the film thickness of each sample, are displayed below the micrographs.

The graphene films were analyzed by atomic force microscopy (AFM) to investigate their morphology and evaluate their thickness (Figure 4). The films were found to uniformly adhere on the Si/SiO_2_ surface. Occasionally, some folded regions can be found, as well as some wrinkles, as expected. The thickness was measured by taking profiles of the films grown at different temperatures. The measured values are in the range of about 1–2 nm, and a monotonic increase in thickness (*t*) with the temperature is observed. All these results are consistent with the analysis of the Raman *I*_2D_/*I*_G_ ratios. These values can be used to estimate the number of layers of the graphene films: 1–2 at 930 °C, 3–4 at 1000 °C, and 6 at 1070 °C.

In Table 1, the optical transmittance of the films is reported. The optical transmittance inversely scales with the CVD temperature, while it is not influenced by the hydrogen flow. The films grown at 930 °C have all an average transmittance of 94% at 550 nm, those grown at 1000 °C have 90%, while the 1070 °C samples have 83%.

**Table 1:** Optical transmittance and thickness (*t*) of pyridine-CVD graphene.

temperature [°C]	*T* @ 550 nm	*t* [nm]	σ_Op_ (·10^5^) S/m

930	0.94	0.9	2.4
1000	0.90	1.3	2.1
1070	0.83	2.1	2.5

The optical conductivity was calculated as *T* = (1 + *Z*_0_/2 σ_Op_·*t*)^−2^, where *Z*_0_ is the impedance of free space (equal to 377 Ω) [54], and *t* is the thickness. The value of *t* at each temperature was set taking in consideration the maximum number of layers as estimated by AFM and the optical transmittance of the film. This is a conservative approach, as the graphene films can occasionally appear thicker when analyzed by AFM in tapping mode [55]. Considering the thickness of a monolayer graphene (equal to 0.335 nm), we have *t*_930 °C_ = 0.9 nm, *t*_1000 °C_ = 1.3 nm, *t*_1070 °C_ = 2.1 nm [16].

**Figure 5:**
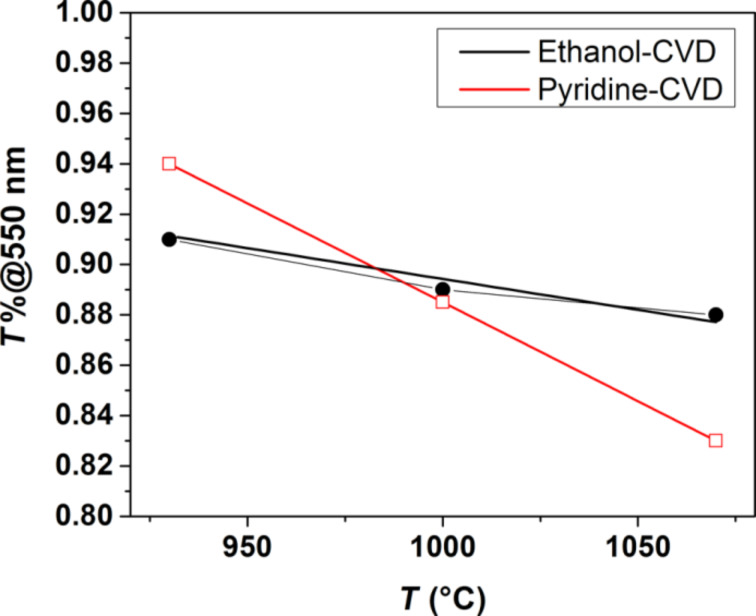
Transmittance vs CVD temperature trends of the graphene films. Data points are provided as red void squares (pyridine-CVD) and black filled circles (ethanol-CVD). The *T*_%_-vs-*T*(°C) dependence can be linearly fitted by *T*_%_ = (1.66−7.8)·10^−4^ *T*(°C) for pyridine-CVD and *T*_%_ = (1.14−2.5)·10^−4^ *T*(°C) for ethanol-CVD.

We plotted the optical transmittance vs the CVD temperature for ethanol- and pyridine-grown films (Figure 5) to gain further insight into the two processes. The optical transmittance at 550 nm and the film thickness were found to be more sensitive to the temperature for the pyridine than for the ethanol case, indicating different kinetics. This effect might be related to the presence of oxygen in the ethanol precursor, which alters the catalytic activity of copper [37,56]. The two liquid precursors could be thus selected to be used at a specific temperature to obtain graphene films of different thickness.

The electrical properties of the graphene films grown by pyridine-CVD are reported in Table 2. The electrical conductivity is linked to the film thickness as *R*_s_ = (σ_DC_·t)^−1^. We have also calculated a figure of merit (FoM) that can be used to compare the electrical/optical properties of thin transparent conductors made from various materials. Such FoM is defined as the conductivity ratio, σ_DC_/σ_Op_ [16].

**Table 2:** Electrical properties of the pyridine-CVD graphene.

	H_2_ flow: 1 sccm				H2 flow: 100 sccm		
			
temperature [°C]	*R*_s_ [kΩ/□]	σ_DC_ [10^5^ S/m]	FoM		*R*_s_ [kΩ/□]	σ_DC_ [10^5^ S/m]	FoM

930	2.4	6	2.5		3.2	4.5	1.9
1000	0.9	8	3.9		0.5	14.3	7
1070	0.65	7.3	3		0.4	11.9	4.8

The temperature is the main parameter dictating the characteristics of the pyridine-CVD graphene films, as also observed for the optical properties and the thickness of the films. At each temperature, the sheet resistance is observed to sweep over a narrow range of values when adding the two different hydrogen flows. Hydrogen seems to lead to a slight decrease in sheet resistance at 1000 and 1070 °C, while this trend is reversed at 930 °C. The electrical characteristics of the pyridine films can be compared to those of ethanol-CVD graphene in Table 3. In this case, the samples with the lowest sheet resistance at each temperature are reported (obtained with specific hydrogen flows, as indicated in the table). As in the case of pyridine-CVD, the optical transmittance decreases with the temperature, as does the sheet resistance.

**Table 3:** Electrical properties of the ethanol-CVD graphene.

temperature [°C]	H_2_ [sccm]	*T*@550 [nm]	number of layers	thickness [nm]	σ_Op_ [10^5^ S/m]	*R*_s_ [kΩ/□]	σ_DC_ [10^5^ S/m]	FoM

930	0	0.91	4	1.3	2	1.8	4.3	2.2
1000	100	0.89	5	1.6	2	0.6	10.4	5.2
1070	10	0.86	6	2.0	2.1	0.5	10	4.8

To account for the intrinsic electrical properties of the graphene films, without reference to the thickness, a study of their electrical conductivity is in order. Overall, the electrical conductivity of graphene from both pyridine and ethanol is observed to increase with the CVD temperature, as expected (and as demonstrated by the progressively lower D peak in the Raman spectra) [30,57]. At 1070 °C, the films show similar thicknesses (around 2 nm) and electrical conductivities (σ_DC_ up to 11.9·10^5^ S/m for the pyridine sample). This points to a negligible effect of doping in the pyridine samples at 1070 °C, as confirmed by Raman and XPS, and as expected at such a high temperature [27].

Upon lowering the temperature to 1000 °C, some differences in the two kinds of graphene films emerge and a weak effect of doping can be detected in the pyridine-derived films (also confirmed by Raman and XPS analysis). With the CVD of ethanol, at 1070 and at 1000 °C pristine graphene films were grown with the same conductivity (σ_DC_ up to 10.4·10^5^ S/m). Instead, in the case of pyridine, the film grown at 1070 °C has a lower conductivity (11.9·10^5^·S/m) than the film grown at 1000 °C (14.3·10^5^ S/m). Upon decreasing the temperature to 930 °C, the electrical conductivity of the samples from both precursors decreases due to the evident defectiveness of the growth at such low temperature (Table S1, Supporting Information File 1). But, the pyridine samples at 930 °C have an electrical conductivity that is 40% higher than that of the ethanol-derived samples (6·10^5^ S/m compared to 4.3·10^5^). This situation strongly supports the occurrence of nitrogen doping during pyridine-CVD below 1000 °C, and in particular at 930 °C, as evidenced previously by the Raman and XPS analysis. Such values of electrical conductivity also demonstrate that the *I*_D/G_ Raman ratios observed in pyridine-derived graphene, which are higher than those of ethanol-derived graphene, are due to quaternary nitrogen doping and not to a more defective graphene lattice. In fact, a defective lattice would rather impair the electrical conductivity making it lower than that of ethanol-CVD graphene [57]. With regard to the FoMs (Table 2 and Table 3), the pyridine samples consistently achieve higher FoMs than the ethanol samples (up to 7 at 1000 °C), suggesting the viable use of pyridine as CVD precursor for the production of efficient transparent conductive electrodes.

The interpretation of the experimental evidence gathered in our analysis can be used to draw some general conclusions about the characteristics of the films grown by pyridine-CVD, in view of their application in electronics. i) The film thickness is directly proportional to the CVD temperature. ii) The electrical conductivity generally improves when increasing the CVD temperature and hydrogen flow, due to a higher graphitization level and to a lower defect density. iii) The electrical conductivity is also linked to the doping level, which is however less pronounced above 1000 °C.

## Conclusion

Graphene films were grown by chemical vapour deposition (CVD) using pyridine as liquid carbon precursor, exploring a range of temperatures between 930 and 1070 °C, and assessing the effect of hydrogen in the CVD gas mixture. A comprehensive range of characterizations (atomic force microscopy, Raman and X-ray photoemission spectroscopy, optical and electrical measurements) was run to ascertain the properties of the graphene samples and the occurrence of doping. These results were compared to the case of pristine graphene films grown by CVD of ethanol, a liquid precursor akin to pyridine. The graphene films grown at high temperatures (1070 °C) are in both cases multilayered with high crystalline quality. Upon lowering the temperature below 1000 °C, the graphene films are on average thinner (down to one or two layers) and with a higher defect density. In particular, at 930 °C significant differences emerge in the Raman spectra of the graphene films grown by the two precursors: the pyridine samples show G, 2D and D band shift which are usually ascribed to n-type doping, while the ethanol samples do not show such trends. XPS spectra confirmed the presence of nitrogen in pyridine-films grown at temperatures below 1000 °C. Graphene grown from pyridine at 930 °C (with 1 sccm of hydrogen flow) had a nitrogen/carbon ratio of 3%, and from the N1s peak analysis the nitrogen atoms appear in quaternary form (i.e., inserted into the graphitic lattice). We conclude that when the CVD temperature is lowered below 1000 °C, non-carbon atoms or groups can insert in the C lattice forming doped graphene. As a result, below 1000 °C the effect of nitrogen doping emerges among these effects and contributes to bring the electrical conductivity of the pyridine samples up to 14.3·10^5^ S/m, i.e., consistently higher values than the pristine samples grown by ethanol-CVD.

## Experimental

### Graphene growth and transfer

Graphene films were grown onto 25 μm thick copper foil (Cu-XLP/PHC Extra low phosphorous copper, 99.95% purity) cut to the desired size and placed over a quartz boat, after cleaning with acetone and ethanol.

Graphene was grown in a low-pressure CVD reactor and the graphene films were transferred onto the substrates of use by a wet transfer method. The reactor consists of a 2 m long, 38 mm inner diameter quartz tube, coaxial to a high temperature furnace. The tube is connected to a rotary vane vacuum pump, a mass flow controlled gas feed system and absolute pressure gauges. The long reactor tube allows the samples to be inserted in and extracted from the heated section without breaking the vacuum or perturbing the gaseous atmosphere. The system design implements a fast cooling scheme allowing several samples to be grown within a single furnace heating cycle, without exposing them to the atmosphere when hot. After the initial ramping of the furnace temperature the pressure was stabilised at 4 mbar by flowing 20 sccm Ar and 20 sccm H_2_. The quartz boat supporting the samples was then inserted into the hot zone and annealed for 1200 s at the growth temperature. Liquid pyridine was contained in a steel “bubbler” vessel pressurized in Ar at 3 bar, which was kept at 20 °C (about 15 mbar equilibrium pressure). After the annealing, the H_2_ gas flow was set to the desired value and the Ar flow was switched from the bubbler vessel at a flow rate of 20 sccm. The amount of vapor entering the chamber was estimated to be 0.5% (15 mbar/3 bar) of the carrier flow, thus 0.1 sccm. After the desired growth time, the samples were extracted from the hot zone, let to cool to near room temperature, and then extracted from the vacuum vessel and further processed for extracting the graphene. When performing the growth with ethanol, the procedure was the same, with the only difference being that the steel “bubbler” vessel in this case contained liquid ethanol (pressurized in Ar at 3 bar) and was kept at 0 °C. It can be noted that the CVD of pyridine requires the same experimental setup as the CVD of ethanol, being both precursors liquid under standard conditions and having similar vapor pressures (about 15 mbar at 20 °C for pyridine and at 0 °C for ethanol).

Graphene was transferred onto 300 nm thermal oxide-coated Si wafers for Raman, XPS, AFM and sheet resistance measurements, and onto glass substrates for optical transmittance. The graphene transfer was performed using a cyclododecane protective layer, a novel method recently developed by our group. Cyclododecane is a waxy solid which sublimates at ambient temperature in a few hours without leaving residues and it was recently demonstrated not to alter Raman nor XPS spectrum of graphene when used, with respects to the free floating method [36]. To further check this point, Raman and XPS spectra were also recorded on samples transferred by free-floating with the same results. The copper foil was etched away by means of an ammonium persulfate bath (PSA, 50g/L) for 3 h at about 20 °C and then transferred in a clean distilled water bath for rinsing. The graphene film was finally scooped out of the distilled water using the destination substrate for its subsequent characterization and use.

### Film characterization

#### Raman spectroscopy

Doping level, degree of sp^2^ crystallinity and mechanical strain were investigated using Raman spectroscopy, with a HORIBA Scientific LabRAM HR Evolution Raman spectrometer with an integrated Olympus BX41 microscope. Laser excitation wavelength of 532 nm (2.33 eV) was focused on the sample surface using a 100× objective with a spot size of approximately 1 μm. Low laser power (below 1 mW) was used to minimize sample heating and possible damages. Because of possible inhomogeneity in the films, the spectra were recorded at ten different spots and averaged.

#### XPS

The electronic and structural properties of the graphene films on Si/SiO_2_ were probed by X-ray photoemission spectroscopy (XPS) with Mg Kα X-ray radiation at 1253.6 eV (VG Escalab MkII Spectrometer). The samples were treated in air at 200 °C for a few minutes before XPS to remove possible organic contaminants.

#### AFM

Graphene-films transferred on silicon have been characterized by tapping-mode atomic force microscopy (using a Bruker-Veeco Dimension Icon AFM). The images were acquired in tapping mode at 0.5 Hz, using Sb-doped Si cantilevers (Bruker) with resonant frequency around 300 kHz. At least five areas for each sample were measured in order to take into account thickness inhomogeneity which might derive from the film transfer (the AFM images shown in Figure 4 are statistically representative of these measurement).

#### Sheet resistance measurement

The electrical sheet resistance of the films transferred to Si/SiO_2_ was measured in a controlled environment under constant temperature (23 ± 0.5 °C) and humidity (35 ± 5%). The test system consisted of a probe with four collinear WC tips (spaced overall by 3 mm), fixed on a stand (Signatone S301) and connected to a current source and a low-voltage meter. The measurements were repeated multiple times over an area of at least 1 × 1 cm^2^ on each sample and then averaged.

#### Optical transmittance

Transmittance spectra at normal incidence have been recorded in the 400–1100 nm wavelength range by a fiber optics spectrophotometer (HR4000CG-UV-NIR model by Ocean Optics), equipped with a tungsten halogen source and connected to the sample stage by quartz optical fibers (1 m length, 600 µm core diameter) with quartz collimating lens mounted on their ends (spot size on the sample about 1 mm). For each sample, the measurement was repeated in three sites to average over film inhomogeneity.

## Supporting Information

In the Supporting Information the results of the Raman spectral fitting for pyridine- and ethanol-CVD are reported.

File 1 Results of the Raman spectral fittings.
