# Supplementary material for: Nitrogen-doped graphene films from chemical vapor deposition of pyridine: influence of process parameters on the electrical and optical properties
Source: Beilstein J Nanotechnol. 2015 Oct 14;6:2028–38. doi: 10.3762/bjnano.6.206 (PMC4660949; doi:10.3762/bjnano.6.206)
Supplement: File 1 — Results of the Raman spectral fittings. [file Beilstein_J_Nanotechnol-06-2028-s001.pdf]

Supporting Information

for

Nitrogen-doped graphene films from chemical vapor deposition of pyridine: influence of process parameters on the electrical and optical properties

Andrea Capasso*[1,2], Theodoros Dikonimos[1], Francesca Sarto[3], Alessio Tamburrano[4], Giovanni De Bellis[4], Maria Sabrina Sarto[4], Giuliana Faggio[5], Angela Malara[5], Giacomo Messina[5] and Nicola Lisi[1]

Address: [1]ENEA, Materials Technology Unit, Surface Technology Laboratory, Casaccia Research Centre,Via Anguillarese 301, 00123 Rome, Italy, [2]Istituto Italiano di Tecnologia, Graphene Labs, I-16163 Genova, Italy, [3]ENEA, Fusion Technical Unit, Lab. of Nuclear Technologies, Via Enrico Fermi 45, 00044 Frascati (Rome), Italy, [4]Research Center on Nanotechnology Applied to Engineering of Sapienza (CNIS), SSNLab, Sapienza, University of Rome, P.le Aldo Moro 5, 00185 Rome, Italy and [5]Dipartimento di Ingegneria dell'Informazione, delle Infrastrutture e dell'Energia Sostenibile (DIIES), Università “Mediterranea” di Reggio Calabria, 89122 Reggio Calabria, Italy

Email: Andrea Capasso* - andrea.capasso@iit.it

*Corresponding author

Results of the Raman spectral fittings

Table S1: Raman properties of pyridine-CVD graphene films.

Samples		Raman parameters									
**H_2**	**T (°C)**	**Γ_D (cm^{-1})**	**ω_D (cm^{-1})**	**Γ_G (cm^{-1})**	**ω_G (cm^{-1})**	**Γ_{2D} (cm^{-1})**	**ω_{2D} (cm^{-1})**	**$I_{D/G}$**	**$I_{2D/G}$**	**$I_{D/D'}$**	**L_a[nm]**
**1 sccm**	**930**	28.1	1343.7	20.0	1585.5	34.5	2682.0	0.92	1.55	3.68	15.26
	**1000**	31.2	1349.6	17.2	1584.3	36.6	2690.7	0.20	0.69	2.51	54.28
	**1070**	32.2	1351.5	16.1	1583.2	37.8	2693.4	0.14	0.6	2.14	73.20
**100 sccm**	**930**	28.7	1344.3	22.5	1586.0	39.4	2682.6	1.16	1.29	4.42	13.31
	**1000**	29.7	1348.2	17.3	1583.2	33.1	2690.8	0.17	0.86	3.19	66.27
	**1070**	33.5	1350.8	17.2	1582.3	40.4	2693.8	0.08	0.75	2.57	124.74

Table S2: Raman ratios of ethanol-CVD graphene films.

Samples		Raman ratios		
**H_2**	**T (°C)**	**$I_{D/G}$**	**$I_{2D/G}$**	**$I_{D/D'}$**
**1 sccm**	**930**	0.68	0.89	3.41
	**1000**	0.13	0.84	2.17
	**1070**	0.05	0.89	1.67
**100 sccm**	**930**	0.25	1.15	2.88
	**1000**	0.19	0.86	3.80
	**1070**	0.06	0.70	2.0
